# A Risk Assessment Framework for the Socioeconomic Impacts of Electricity Transmission Infrastructure Failure Due to Space Weather: An Application to the United Kingdom

**DOI:** 10.1111/risa.13229

**Published:** 2018-11-08

**Authors:** Edward J. Oughton, Mike Hapgood, Gemma S. Richardson, Ciarán D. Beggan, Alan W. P. Thomson, Mark Gibbs, Catherine Burnett, C. Trevor Gaunt, Markos Trichas, Rabia Dada, Richard B. Horne

**Affiliations:** ^1^ Environmental Change Institute University of Oxford Oxford UK; ^2^ RAL Space, Science and Technology Facilities Council Harwell UK; ^3^ British Geological Survey Edinburgh UK; ^4^ Met Office Exeter UK; ^5^ University of Cape Town Cape Town South Africa; ^6^ Airbus Defence and Space Stevenage UK; ^7^ University of Cambridge Cambridge UK; ^8^ British Antarctic Survey Cambridge UK

**Keywords:** Critical infrastructure failure, socioeconomic impact assessment, space weather

## Abstract

Space weather phenomena have been studied in detail in the peer‐reviewed scientific literature. However, there has arguably been scant analysis of the potential socioeconomic impacts of space weather, despite a growing gray literature from different national studies, of varying degrees of methodological rigor. In this analysis, we therefore provide a general framework for assessing the potential socioeconomic impacts of critical infrastructure failure resulting from geomagnetic disturbances, applying it to the British high‐voltage electricity transmission network. Socioeconomic analysis of this threat has hitherto failed to address the general geophysical risk, asset vulnerability, and the network structure of critical infrastructure systems. We overcome this by using a three‐part method that includes (i) estimating the probability of intense magnetospheric substorms, (ii) exploring the vulnerability of electricity transmission assets to geomagnetically induced currents, and (iii) testing the socioeconomic impacts under different levels of space weather forecasting. This has required a multidisciplinary approach, providing a step toward the standardization of space weather risk assessment. We find that for a Carrington‐sized 1‐in‐100‐year event with no space weather forecasting capability, the gross domestic product loss to the United Kingdom could be as high as £15.9 billion, with this figure dropping to £2.9 billion based on current forecasting capability. However, with existing satellites nearing the end of their life, current forecasting capability will decrease in coming years. Therefore, if no further investment takes place, critical infrastructure will become more vulnerable to space weather. Additional investment could provide enhanced forecasting, reducing the economic loss for a Carrington‐sized 1‐in‐100‐year event to £0.9 billion.

## 1. INTRODUCTION

Space weather can cause direct disruption to critical national infrastructure (CNI), including electricity transmission, satellite communications and global navigation satellite systems, aviation, and rail transportation (Riley et al., 2018). Cascading failure can indirectly lead to the disruption of other essential systems. Space weather forecasting is essential to ensure CNI operators have time to implement operational mitigation measures to protect critical systems, reducing potential negative consequences. Yet, evidence on our shared global vulnerability to space weather, the potential socioeconomic impacts of CNI service disruption, and the effect of different forecasting capabilities is still limited, despite this being essential (Schrijver et al., 2015). Internationally, there is now a new push to develop space weather mitigation strategies, especially in North America and Europe, as illustrated by President Obama's 2016 Executive Order (White House, 2016) or the U.K. Space Weather Preparedness Strategy (Cabinet Office & Department for Business, Innovation & Skills, 2015). This has prompted the need for increased risk analysis of space weather threats (North, 2017).

Space weather includes multiple solar eruptive phenomena, including coronal mass ejections (CMEs), solar energetic particles, and bursts of electromagnetic radiation (also known as “solar flares”). We focus here on the impact of CMEs, consisting of billions of tons of electrically charged particles, carrying a magnetic field, ejected from the Sun into the interplanetary space (Webb & Howard, 2012). Extreme geomagnetic “storms” can arise when large (10^12 ^kg), dense (100 particles/cm^3^), and fast (>500 km^−1^) CMEs couple with Earth's magnetic field, particularly when the CME carries a significant southward‐pointed direction (“B_z_”) magnetic field (Balan et al., 2014; Möstl et al., 2015; Temmer & Nitta, 2015). One significant terrestrial impact of space weather is that it drives large geomagnetic storms and their associated magnetospheric “substorms,” which produce intense and rapidly varying ionospheric currents. The generation of geomagnetically induced currents (GIC) that follows from such rapid changes in Earth's magnetic field can pose a risk to the electrical power transmission network as GIC flow from and to grounding points at transmission substations, leading to the partial saturation of transformers (Boteler & Pirjola, 2014; Kappenman, 1996; Molinski, 2002; Viljanen & Pirjola, 1994).

While there has been considerable research published in the scientific peer‐reviewed literature on the likelihood and severity of space weather phenomena, few studies have undertaken rigorous and robust quantification of the socioeconomic impacts of space weather (Eastwood et al., 2017). This has left many scientists and other risk analysts feeling dissatisfied with the level of analysis presented in the gray literature. Our contribution is to provide a method that overcomes some of the limitations of previous analyses (Oughton et al., 2016; Oughton, Skelton, Horne, Thomson, & Gaunt, 2017; Schulte in den Bäumen, Moran, Lenzen, Cairns, & Steenge, 2014). This includes properly capturing (i) geophysical risk resulting from combined space and solid Earth physics, (ii) properties of infrastructure assets, and (iii) the network structure of the high‐voltage power grid. This information is then used to quantify the potential socioeconomic impacts of space weather due to failure in electricity transmission, under different space weather forecasting capabilities. Both short‐term power outages due to voltage instability, and long‐term power outages due to transformer damage from thermal heating, are considered.

The research questions we investigate include:

1. What is the probability of CNI being affected by intense magnetospheric substorms?
2. How vulnerable are specific electrical transmission CNI assets and nodes to GIC exposure during intense substorms?
3. What are the potential socioeconomic impacts of electrical transmission CNI failure due to space weather, under different forecasting capabilities?

In Section 2, a literature review is undertaken. In Section 3 the method is articulated, with the results being presented in Section 4 and discussed in Section 5. Final conclusions are provided in Section 6.

## 2. LITERATURE REVIEW

Space weather is a high‐impact, low‐frequency (HILF) event. One of the most notable geomagnetic storms is known as the “Carrington Event” of September 1859 and has been the focus of many scientific studies (e.g., Boteler, 2006; Green & Boardsen, 2006; Ribeiro, Vaquero, & Trigo, 2011; Saiz, Guerrero, Cid, Palacios, & Cerrato, 2016; Silverman, 2006; Siscoe, Crooker, & Clauer, 2006; Tsurutani, Gonzalez, Lakhina, & Alex, 2003). However, data from this period are limited, giving rise to considerable diversity in the estimates of the size of the event. Within the digital age, the two key events studied include the March 1989 and October–November 2003 severe magnetic storms. During the severe 1989 geomagnetic disturbance (GMD), the Hydro‐Quebéc power grid experienced a voltage collapse, leaving 6 million customers without power for almost nine hours before the supply was restored (Barnes & Dyke, 1990). In July 2012, a very large and fast CME was observed by spacecraft but missed Earth. Estimates indicate this storm could have been Carrington‐sized had it hit Earth (Baker et al., 2013).

### 2.1. Frequency and Severity

Geomagnetic activity is often studied using extreme value statistics (Lotz & Danskin, 2017; Rodger et al. 2017; Thomson, Dawson, & Reay, 2011). However, there are limited time‐series data on which to understand both the frequency and severity of large events (Hapgood, 2011). Therefore, with only a limited catalogue of actual events, analysts often rely on extrapolations of power law or lognormal distributions to estimate extremes. For example, Riley and Love (2017) estimate the probability of an extreme event comparable to Carrington taking place in the next decade is 10.3% using a power law distribution and 3% using a lognormal distribution. Analysis by Kataoka (2013) estimates the probability of occurrence of extreme geomagnetic storms as a function of the maximum sunspot number of a solar cycle, with the probability of a Carrington‐sized storm being 4–6% over the next decade. Jonas, Fronczyk, and Pratt (2018) apply a Bayesian model average to the estimates of Riley (2012), Roodman (2015), and Love, Rigler, Pulkkinen, and Riley (2015) to develop probabilities of space weather events of different intensities, finding an estimated 37% likelihood for an event comparable with March 1989 over a 10‐year period. Due to data limitations, estimates for a Carrington‐sized event are far more uncertain, ranging from approximately 1% to 10% over a 10‐year period. Finally, Thomson et al. (2011) assess horizontal geomagnetic field changes, finding that a typical mid‐latitude (55–60° north) European observatory may experience a one‐minute peak rate‐of‐change in the field reaching 1,000–4,000 (1,000–6,000) nanoteslas per minute (nT/min) once every 100 (200) years (additional information in Section 3.1 and Supporting Information Appendix A).

### 2.2. The Impact of GIC on Electricity Transmission Infrastructure

GICs are correlated with and well characterized by the time derivative (rate‐of‐change, *dH/dt*) of the horizontal component (*H*) of the magnetic field (Bolduc, Langlois, Boteler, & Pirjola, 1998). Effective parameterization and prediction of GIC is challenging, requiring information on ground conductivity and magnetic field variations in relation to the exposed power grid structure (Boteler, 2014). Comprehensive analyses of the current understanding of space weather GIC hazards to power grids can be found in Gaunt (2016) and Pulkkinen et al. (2017).

The most significant effects of GICs on power systems derive from the nonlinear magnetic core response of a transformer to GIC (Bolduc, Gaudreau, & Dutil, 2000; Boteler, Shier, Watanabe, & Horita, 1989). As the core is driven into partial saturation by the low‐frequency GIC (with effects similar to those of direct current), the transformer exhibits some of the characteristics of an inductor or reactor in the power circuit; the reactive power drawn by the transformer increases (approximately in proportion to its power rating and the GIC present) and a power frequency current higher than the normal current flows in the transformer, with three main effects. First, the heat “generated” by losses inside the transformer causes its temperature to rise (Marti, Rezaei‐Zare, & Narang, 2013), even to the extent of initiating damage to the winding conductors or paper insulation or the breakdown of the oil, with the result that the automatic protection removes (trips) the transformer from the system. Second, the increased current causes the voltage drop in all lines to increase, possibly to the point that the voltage cannot be sustained by the automatic tap changers on the transformers, and the system switches off to protect itself from the abnormally low voltages and high currents. As the voltages fall, the effectiveness of shunt capacitors (used for voltage support) falls too, so the response of the system to the GIC‐reactive power combination is, again, not linear, potentially leading to voltage collapse. Finally, the increased current, which has a high harmonic content because of a transformer's nonlinear response, can trip an overcurrent protection relay, or the harmonics may cause the correct or incorrect operation of other types of relays, removing important components from the system, including lines and shunt capacitor banks. These protection relay operations, including the tripping of a damaged transformer, can cause localized loss of supply and aggravate the possibility of voltage collapse (Albertson, Thorson, & Miske, 1974). In addition to these effects, the harmonic distortion propagates into the distribution networks and can affect negatively the performance of customers’ electrical and electronic equipment (Schrijver, Dobbins, Murtagh, & Petrinec, 2014).

### 2.3. Space Weather Socioeconomic Impacts

The key dimensions of the literature on the socioeconomic impacts of space weather have been highlighted in Table I. We particularly emphasize whether different studies include data‐derived ground conductivity risk, asset vulnerability, and network structure because this has generally been a limitation.

**Table I:** Literature Review of Existing Space Weather Impact Assessments

	Geography		Spatio‐Temporal Impacts						Economic Impact				
References	Country	Region	Population Affected	Restoration Period	Ground Conductivity Data?	Asset Vulnerability?	Explicit Network Structure?	Economic Method	Asset Damage	Direct Economic Impact	Indirect Economic Impact	Total Economic Impact	Formally Peer Reviewed?
Barnes and Dyke (1990)	USA	North East	Not stated	50% connected in 16 hours, 75% in 24 hours, 100% in 48 hours	No	No	No	Value of lost load estimation	$16 million (1988 USD)	$3–6 billion (1988 USD)	Not modeled	Not modeled	Yes
Bolduc (2002)	Canada	Quebec	9 million	N/A	No	No	No	Not stated	$13.2 million (CAN dollars)	Not modeled	Not modeled	Not modeled	Yes
Pulkkinen, Lindahl, Viljanen, and Pirjola (2005)	Finland	Malmö	50,000	One hour	No	No	No	Not stated	Not stated	$0.5 million (USD)	Not modeled	Not modeled	Yes
Kappenman (in Space Studies Board, 2008)	USA	National	Not stated	4 to 10 years	Yes	Yes	Yes	Not stated	Not stated	$1–2 trillion (USD)	Not stated	Not stated	No
Lloyd's of London (2013)	North America	N/A	20–40 million	16 days to one to two years	Yes	Yes	Yes	Value of lost load estimation	Not stated	$0.6–2.6 trillion (USD)	Not modeled	Not modeled	No
Schulte in den Bäumen et al. (2014)	Global	National	Not stated	Five months to one year	No	No	No	Multi‐regional input–output analysis	Not modeled	Not stated	Not stated	$3.4 trillion (USD)	Yes
Schrijver et al. (2014)	North America	National assessment	N/A	N/A	N/A	N/A	N/A	Retrospective cohort exposure study	Not stated	∼4% of claims are statistically associated with geomagnetic activity	Not modeled	Not modeled	Yes
PwC (2016)	Europe	N/A	“3 cities”	Three days	No	No	No	Input–output analysis	€0.26–0.31 billion	€2–2.7 billion	€1.7–2.1 billion	€3.7–4.8 billion	No
Oughton et al. (2017)	USA	National assessment	8–66%	24 hours	No	No	No	Multi‐regional input–output analysis	Not modeled	$3–28.2 billion (USD)	$1.4–7.2 billion (USD)	$4.4–35.4 billion (USD)	Yes
ABT Associates (2017)	North America	National assessment			No	No	No	Value of lost load estimation	Not stated	Not stated	Not stated	∼$0.4–10 billion (moderate), $1–20 billion (extreme)	No

A frequently referenced study by Lloyd's of London (2013) assesses the risk to the North American electricity grid, estimating that the potential total cost for a scenario where 20–40 million people were left without power for 16 days to 1–2 years, could range from $0.6 to $2.6 trillion USD. In a cost‐benefit analysis of the European Space Agency's (ESA) Space Situational Awareness program, PricewaterhouseCoopers (PwC, 2016) estimated the gross domestic product (GDP) impact of a space‐weather‐induced blackout to be approximately €5.7 billion, predicated on a three‐day blackout taking place in three major European cities.

Within the peer‐reviewed literature, Schulte in den Bäumen et al. (2014) analyzed the global consequences of severe space weather on East Asia, Europe, and North America, finding that a Quebec‐1989‐like event could see a global economic impact of $2.4–3.4 trillion over a year, leading to a global GDP loss of 3.9–5.6%. In a study focusing just on the United States, Oughton et al. (2017) estimated the daily loss from electricity transmission failure for the United States based on different geomagnetic storm footprints, finding that it could range from $7 to $42 billion.

Very few studies have assessed the potential ramifications of space weather forecasting. One rare example by Teisberg and Weiher (2000) finds that the net benefits of a satellite warning system are strongly positive, and having undertaken a sensitivity analysis, remain positive even if the damage is as low as $2 billion. Enhanced space weather forecasting capability has the potential to (i) increase the warning time prior to an event taking place, and (ii) increase the confidence in the forecast, reducing the probability that the warning will be ignored (for a discussion of the cost‐loss implications of space weather forecasting, see Henley & Pope, 2017). Three key actions that can be enabled include implementation of infrastructure operator mitigation plans, business continuity plans, and local building and community resilience activities. The key action in this case is the ability for CNI operators to engage emergency mitigation plans earlier, helping to prevent both damage to key assets and potential loss of human life following CNI disruption.

## 3. METHOD

**Figure 1:**
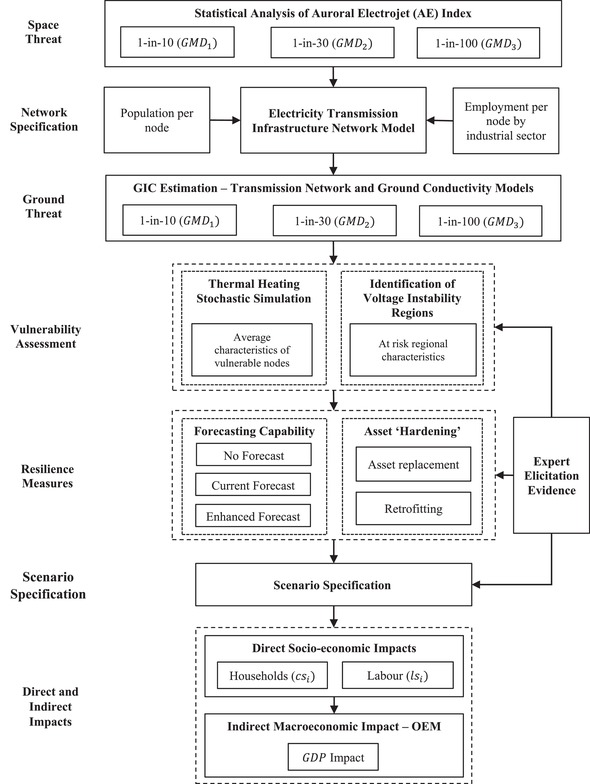
Assessment framework.

A general assessment framework is developed for the United Kingdom. We test specific *GMDs*, which are a threat to the system of study, referring to different variations as ${\mathrm{GMD}}_{1},\ldots ,{\mathrm{GMD}}_{z}$, with each scenario representing a different level of threat manifestation (1‐in‐10‐year, 1‐in‐30‐year, and 1‐in‐100‐year). Specifically, in our study scenario $GMD_{i}$ signifies that during event *i*, *m* extra high voltage (EHV) transformers (≥275 kV) in a transmission substation node (*n*) within the network could have failed due to GIC exposure. Hence, each *n* node contains multiple transformers $m_{1},\ldots ,m_{z}$, with each transformer having a set of technical characteristics $c_{1},\ldots ,c_{z}$, indicating that each transformer type has a different level of vulnerability to GIC. Thus, for a comprehensive vulnerability assessment of each space weather event $GMD_{i}$ we simulate failure possibilities in the system, giving rise to a set of failure scenarios $S_{1},\ldots ,S_{d}$. The impact of each scenario is initially measured in terms of the proportion of directly affected consumers $cs_{1},..,cs_{i}$ and directly affected labor $ls_{1},..,ls_{i}$ at each node. Consequently, the level of disruption is estimated based on electricity loss for a set of event scenarios $S_{1},..,S_{h}$ and is quantified using lost GDP. Fig. 1 illustrates the framework applied to the United Kingdom.

### 3.1. Space Threat

We construct GMD scenarios that are time sequences of substorms of differing intensities. These sequences are based on the auroral electrojet (AE) geomagnetic activity index measured in nanoteslas (nT) (Davis & Sugiura, 1966), sourced from the U.K. Solar System Data Centre. We use data from October 28 to 29, 2003 to construct a 1‐in‐10‐year scenario ($GMD_{1})$ and March 13–14, 1989 to construct a 1‐in‐30‐year scenario ($GMD_{2})$. In each case we smoothed the AE data by taking a 31‐minute running median (to suppress short‐lived spikes in the data), and identify substorms as distinct peaks in the smoothed data. We focus on the most intense peaks with AE > 1,500 nT, as only these are considered to have potentially significant impacts, and we describe these peaks as “very intense substorms.” For the purposes of this study, we use a conservative assumption that the potential impact maximizes if the substorm occurs around 01:00–03:00 local time at the grid location. This is consistent with the voltage collapse of the Quebec grid (Bolduc, 2002), which occurred during a very intense substorm around 03:00 local time on March 13, 1989. Supporting Information Appendix A provides a detailed methodological note on this procedure.

To construct a 1‐in‐100‐year scenario ($GMD_{3})$, data are adapted from the 1989 storm to match key features of the Carrington event of 1859, which comprised two geomagnetic storms, (i) a very large storm with a sudden storm commencement (SSC) around 05:00 UTC on September 2, preceded by (ii) a smaller but still large storm with an SSC around 22:30 UTC on August 28 (Stewart, 1861). These adaptations shift the SSC to the correct time of day and year, the former being the key change for the purposes of our analysis since, as discussed above, it determines when a power grid is in our risk window of 01:00–03:00 local time. Thus, to represent the September 2 storm, the 1989 AE time series is time shifted so that the SSC in that series moves from 01:27 UTC on March 13 to 05:00 on September 2. Additionally, AE values are added to represent the August 28 storm using another copy of the 1989 AE time series, but instead time shifted so that the 1989 SSC moves to 22:30 on August 28. We then overlay this subset, without any scaling, onto the first. The net result is a time series of simulated AE values covering 15 days around the Carrington event and including variations that we can consider representative of the two large storms recorded by Stewart (1861). We then apply median smoothing and thresholding, as above, to derive a sequence of substorms that we use as our 1‐in‐100‐year scenario ($GMD_{3})$.

We map the 1‐in‐100‐year scenario into grid impacts by assuming, as above, that this maximizes where the local time is 01:00–03:00 at the time of the substorm. This leads to major impacts in North America, consistent with the many reports that the Carrington event generated intense aurora over North America (Green & Boardsen, 2006). It also generates major impacts in Australia, New Zealand, Japan, China, and parts of Russia. However, it does not generate very severe impacts over Western Europe, due to the SSC timing matching the Carrington event. The scenario is expanded to consider a full 24‐hour range of SSC times to reflect CMEs arriving at Earth at different times of day. This is achieved by varying the SSC in one‐hour steps from 0 to 23 hours and varying the footprints westward by 15° at each step. Thus, we generate 24 different scenarios for each risk level and can estimate how many lead to very intense substorms over the United Kingdom. The results offer evidence for each return period to answer the first research question, as well as provide contextual information to inform the scenario specification.

### 3.2. Electricity Transmission Infrastructure Network Model

**Figure 2:**
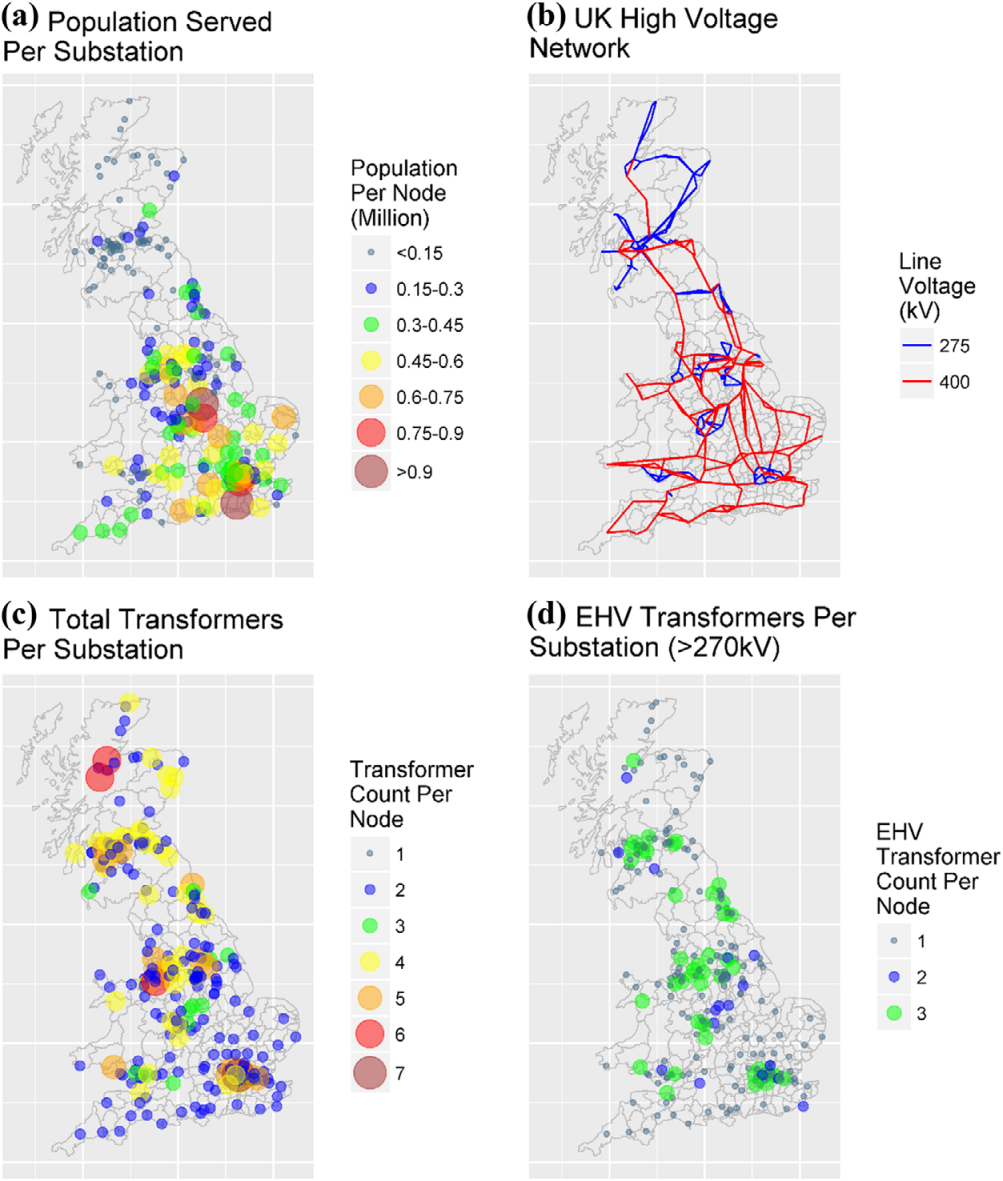
(a) Population served per substation, (b) British high‐voltage network, (c) total transformers per substation, and (d) EHV transformers per substation.

The British high‐voltage transmission power grid consists of a 275 kV and 400 kV transmission network (we exclude the higher resistance 132 kV Scottish lines). A detailed description of the British high‐voltage power network is developed using public information from the National Grid Electricity Ten Year Statement released in 2016, augmented by an extensive search of online maps and satellite imagery. This network model consists of latitude, longitude, and certain electrical characteristics (earthing, transformer, and line resistances) of each substation node and line in the network; the 2016 model has 307 grounded nodes and 519 lines. Some connections are very short, for example, between two transformers on the same site, while the longest is 189.5 km. The median line length is 15 km (mean: 22 km). In the absence of a local distribution network model, we affiliate the local population to the nearest grid node, as illustrated in Fig. 2(a). The structure of the high‐voltage networks for Britain is illustrated in Fig. 2(b), along with the total and EHV‐only transformer assets per node (Figs. 2(c) and 2(d), respectively).

The statistical data from the Office for National Statistics (ONS, 2016) are used in this process, consisting of 7,201 middle output areas for England and Wales, and 1,279 intermediate data zones for Scotland. Hence, this leads to a total of 8,480 statistical areas. Employment data are also obtained via the open‐access Business Register and Employment Survey, and simplified from 18 broad industrial groups to nine.

### 3.3. Ground Threat

To generate a realistic representation of the spatial variation of the geomagnetic field during a large storm, a model of the largest digitally measured magnetic field events (October 2003, for the 1‐in‐10‐year storm, and March 1989, for the 1‐in‐30‐year, or rarer, storm) is constructed based upon measurements from five geomagnetic observatories. Supporting Information Appendix B provides a detailed method on the GIC estimation and validation procedures utilized.

Using (scaled) magnetic field data and a U.K.‐specific Earth conductivity model, a map of the geoelectric field is generated for every minute of the two events. This is then combined with the electricity transmission infrastructure network model to determine GIC per node. If the ground resistance is sufficiently high, the low‐resistance wires of the network offer an easier route for GIC to pass through the earthed neutrals of the connecting transformers, essentially creating a short circuit. In some cases, there are insufficient data to determine the earthing resistance, so we have assumed this to be 0.5 Ω (Kelly, Viljanen, Beggan, & Thomson, 2017). These network parameters are used to calculate GIC (in amperes) along power transmission lines according to Lehtinen and Pirjola (1985):

(1) $$I={\left(1+Y\cdot Z\right)}^{-1}\cdot J,$$

where *J* is the “perfect earthing” current (the geo‐voltage computed between nodes divided by the connecting line resistances), *Z* is the impedance matrix, *Y* is the network admittance matrix, and *I* is the vector containing the estimated GIC at each node. The input data from the network parameters are used to calculate *Y* and *Z*. The geo‐voltage for *J* is calculated by interpolating the electric field grid value onto the power transmission lines and integrating along the line. The GIC at each node on the grid are then computed, calculated from the sum of both the North and East components of the surface electric field. The method for the calculation of GIC in the British network has been benchmarked against Horton, Boteler, Overbye, Pirjola, and Dugan (2012) in Richardson and Beggan (2017), by ensuring that the GIC values produced by the model code are consistent for the given grid, and thus are valid.

The Dst index is a measure of the severity of a GMD based on ring current intensification. For the 1‐in‐10‐year event we use the Dst magnitude for 2003 (−383 nT) (Echer, Gonzalez, & Tsurutani, 2008) ($GMD_{1})$ and for the 1‐in‐30‐year ($GMD_{2})$ we use 1989 (−589 nT) (Allen, Sauer, Frank, & Reiff, 1989). These return periods are supported by Jonas et al. (2018). Since we expect more intense auroral currents in larger GMDs, GIC values from 1989 may be scaled to reflect more severe events. The Dst of the Carrington event was originally estimated as −1,760 nT (Tsurutani et al., 2003). This estimate was based on a short‐duration (half‐width ∼40 minutes) dip of 1,600 nT in the horizontal magnetic field intensity measured at Mumbai during the 1859 storm. Siscoe et al. (2006) reanalyzed these data using standard procedures for Dst derivation, for example, hourly averaging, and derived a value of −850 nT for the minimum Dst of the Carrington event. This latter value has now been widely adopted in assessments of the Carrington event (e.g., Pulkkinen, Bernabeu, Eichner, Beggan, & Thomson, 2012; Riley & Love, 2017), and produces a scaling factor of ×1.4 for a 1‐in‐100‐year event ($GMD_{3})$, though Cliver and Dietrich (2013) propose a range from −850 to −1050 nT. In this article, we take a conservative approach and use the −850 nT value derived by Siscoe et al. (2006). There is a level of uncertainty in these estimates, as reflected in Jonas et al. (2018); however, exploring further event sizes is beyond the scope of this article.

For the vulnerability analysis, we also include additional extreme scenarios for exploratory purposes. This also helps emphasize the uncertainty arising from extrapolating Dst and the return time. Following Jonas et al. (2018, p. 4), a 1‐in‐500‐year event is estimated to correspond to Dst of −1,400 nT, producing a scaling factor of ×2.4, and a 1‐in‐1,000‐year event has an approximate Dst of −1,800 nT, resulting in a scaling factor of ×3.1. Finally, Vasyliūnas (2011) proposes a *theoretical upper limit* for the largest geomagnetic storm possible corresponding to a Dst of −2,500 nT, which we utilize as an example of equivalent to a “1‐in‐10,000‐year” event, with a scaling factor of ×4.2.

### 3.4. Vulnerability Assessment

In this section, we describe the method for undertaking a vulnerability assessment of transmission infrastructure assets and nodes to thermal heating and voltage instability. Regarding thermal heating, we develop a stochastic simulation model whereby the probability of transformer failure scales based on GIC exposure per transformer. Failure is defined as the complete interruption of part of the electricity system.

Two routes to failure are considered. First, transformer damage can result from thermal heating, which subsequently prevents the asset carrying out normal functionality, requiring an inspection and component replacement, or potentially replacement with an onsite or offsite spare prior to reconnection. This route to failure represents a long‐term power outage, whereby assets affected in this way take weeks to restore to normal service. Second, automatic or manual protection removes transformer assets from active operation as a result of voltage instability, requiring a physical inspection before being returned to service. This route to failure represents a short‐term power outage, whereby assets affected in this way can be returned to service in a matter of hours or days. We assume that the instantaneous peak GIC per node is of sufficient amplitude and temporal duration to cause transformer asset failure during each intense substorm. The results generated are utilized in the scenarios tested later in this article.

After the severe 1989 space weather event, the U.K. transmission grid operator introduced improved hardening measures for new transformer assets capable of being exposed to higher GIC exposure thresholds, commencing with transformer procurements made in 1999. However, due to the asset replacement cycle taking multiple decades, these protections are not yet fully pervasive. Data on the transformer fleet characteristics (including high and lower voltage‐side resistances and earthing arrangements) are commercially sensitive and hence unavailable for this analysis. Therefore, we reflect these existing asset‐hardening measures by exploring the sensitivity of transformer failure based on the random allocation of this unknown parameter. Expert elicitation interviews with the operator provided information regarding four heterogeneous transformer types, each with a different set of technical characteristics, in which 50% are *c*_1_ and can withstand 200 A of GIC in the neutral, 25% are *c*_2_ and can withstand 100 A, 12.5% are *c*_3_ and can withstand 50 A, and 12.5% are *c*_4_ and can withstand 25 A. The probability of failure $p_{i}$ for each transformer is thus scaled between the lower withstand threshold and a threshold 100 A above, based on the GIC for the *m* transformer at each *n* node. Using the following scaling equation yields 0 ≤ $p_{i}$ ≤ 1 for transformer design characteristic types $c_{1},\ldots ,c_{z}$:

(2) $$p_{i}=\frac{{\mathrm{GIC}}_{i}-\min\left(c_{z}\right)}{\max\left(c_{z}\right)-\min\left(c_{z}\right)}.$$

The results of this simulation provide a distribution based on the frequency of transformer failures. We also examine the frequency of *n* node failures, assuming this takes place if more than half of the *m* EHV transformers present fail. The simulation is run for 1,000 iterations and the resulting distributions on transformer and node failures provide average population and employment characteristics per node under each scenario.

Regarding voltage instability, we again utilize expert elicitation methods to identify zones at risk. Voltage instability is stated as being the most likely impact to the U.K. grid (Cannon et al., 2013). When large GICs enter and exit power transmission systems this phenomenon can cause a variety of reported problems, including reactive power surges and system voltage dips leading to grid instability (Boteler et al., 1989). If the GICs produced are large enough, the system can no longer handle the reactive power being demanded, causing voltage collapse and a system‐wide power outage (Hutchins & Overbye, 2011). The system operator considers the largest voltage instability risks to be present in key urban conurbations. This is due to the density of transformer assets and the losses associated with transmitting reactive power over long distances.

### 3.5. Resilience Measures

There are a variety of resilience measures for space weather threats, including both forecasting and infrastructure “hardening.” Some asset hardening has already taken place in the British grid and is partially reflected in the vulnerability analysis articulated in the previous section via heterogeneous asset exposure thresholds. However, retrofitting existing transformers is expensive and not being contemplated at this stage in the United Kingdom. Hence, the emphasis of this article is on U.K. space weather forecasting because it is a recognized resilience measure, enabling the advanced implementation of operational mitigations. This includes increasing generation capacity and reducing power transfer in heavily loaded lines (Bolduc, 2002).

Increasing total system generation capacity reduces generation output below each specific transformer's rated level of operation, reducing normal operational heating and providing more capacity to absorb abnormal GIC exposure. This may be adequate to avoid GICs causing or initiating permanent damage to a transformer. Decreasing power transfer on transmission lines reduces line voltage drop and diminishes the risk of being exposed to voltage “instability” or a classic voltage collapse of the power system. As the level of forecasting capability has a significant impact on our subsequent ability to deal with this risk, we therefore describe how this differs by scenario, using evidence gathered via expert elicitation in collaboration with the U.K. Met Office's Space Weather Operations Centre (MOSWOC). Supporting Information Appendix C provides a detailed overview of space weather forecasting capabilities.

In a *no forecast* scenario, existing space weather satellite observing systems are not replaced prior to the end of their operational life or the scientific mission for which they were originally intended, leaving no coronagraphs available from the Solar and Heliospheric Observatory (SOHO) or the Solar Terrestrial Relations (STEREO) assets. This significantly reduces the forecasting capability and may in extremis render forecasting of severe space weather events useless. At present there are plans under consideration in the United States that may lead to SOHO, the Deep Space Climate Observatory, and the Solar Dynamics Observatory being replaced by a mixture of operational and science missions, but no commitment has been made yet. Similarly, while planning is in progress within ESA, a decision on whether to replace the off Sun–Earth line, side‐on view currently provided by STEREO is not expected until the end of 2019 at the earliest. Unlike STEREO, this mission would be permanently located close to the Lagrange L5 point, 60 degrees behind the Earth in its orbit. This is the optimal view for operational purposes as it enables continuous monitoring of solar active regions just before they are positioned to launch CMEs toward Earth.

The *current forecast* scenario reflects the present forecasting capability, based on existing satellites, allowing forewarning of active regions on the Sun (three to four days before CME arrival). Once a CME has launched SOHO and STEREO coronagraphs are available to support CME forecast arrival time within ±6 hours, the nonoperational status of the spacecraft data results in delayed recognition of the potential threat level. Data gaps degrade the reliability and accuracy of forecasts.

The *enhanced forecast* scenario reflects the standard that could be achieved if the current observations were supplemented by satellites on and off the Sun–Earth line with dedicated L1 and L5 spacecraft. This would allow a longer (six to seven days) forewarning of the complexity of an active region. Coronagraphs, combined with an improved assessment of background solar wind, would provide a much higher level of confidence in the CME arrival time (±4 hours). Moreover, a heliospheric imager would allow updates to be made to the arrival time during CME transit. Table II provides a detailed behavioral description for different forecasting capabilities by scenario.

**Table II:** Detailed Description of Space Weather Forecasting Capability by Scenario

	No Forecast	Current Forecast	Enhanced Forecast
1‐in‐100 year	Very challenging to discriminate between a minor event and a significant 1‐in‐100‐year event. Therefore, infrastructure operators do not have sufficient confidence to implement operational mitigations.	Infrastructure operators implement their currently agreed operational mitigations. While this can help to partially mitigate the risk, poor confidence/accuracy in the forecast means mitigation is likely to be suboptimal due to the associated cost of implementation.	The early identification of a complex active region allows infrastructure operators to fully implement a wider range of operational mitigations. Additional confidence in the arrival time increases the perception of the threat, providing a clearer cost/benefit ratio for operational mitigations. This partially results from a lower “false alarm” rate.
1‐in‐30 year	Very challenging to discriminate between a minor event and a significant 1‐in‐30‐year event. Unless there was evidence that it might be extreme, we assume that infrastructure operators decide not to implement operational mitigations.	Infrastructure operators do not fully implement currently agreed operational mitigations due to the expected levels of severity. While this can help to partially mitigate the risk, poor confidence/accuracy in the forecast means mitigation is likely to be suboptimal due to the associated cost of implementation.	The additional lead time in identifying a complex active region allows National Grid longer to implement a wider range of mitigating actions. Additional confidence in arrival time increases the perception of the threat, providing a clearer cost/benefit to mitigating actions. This partially results from a lower “false alarm” rate.
1‐in‐10 year	Very challenging to discriminate between a minor event and a significant 1‐in‐10‐year event. Unless there was evidence that it might be extreme, we assume that infrastructure operators decide not to implement operational mitigations.	Infrastructure operators do not fully implement currently agreed operational mitigations due to the expected levels of severity. At this scale of event, it is envisaged that the risk would be effectively mitigated, resulting in only minor impacts.	The early identification of a complex active region allows infrastructure operators to fully implement a wider range of operational mitigations. Additional confidence in the arrival time increases the perception of the threat, providing a clearer cost/benefit ratio for operational mitigations. This partially results from a lower “false alarm” rate.

In both the current forecast and enhanced forecast capabilities, satellite missions need to be accompanied by investment to ensure computer models, systems, and staff are in place to predict and communicate space weather.

### 3.6. Scenario Specification

Scenario analysis is a foresight tool that enables the testing of exogenous shocks to a system of study. This technique enables the production of comparative analytics that support strategic decision making. For a review of scenario approaches for risk analysis, see Tosoni, Salo, and Zio (2017). Where gaps exist in specifying scenario parameters because traditional scientific analysis is infeasible or not yet available, we utilize expert elicitation. This was undertaken based on information obtained from a co‐organized workshop, and a set of detailed stakeholder interviews conducted with organizations responsible for critical infrastructure and associated risk management activities. The Space Weather and Critical Infrastructures workshop was held in Ispra, Italy (see Krausmann, Andersson, Gibbs, & Murtagh, 2016), co‐organized with Europe's Joint Research Centre, the Swedish Civil Contingencies Agency, the U.K. Met Office, and the U.S. NOAA's Space Weather Prediction Centre. Expert elicitation interviews were targeted with leading individuals across energy (4), aviation (2), transportation (2), satellite (3), insurance (4), government (9), and academia (4) to assess current exposure to space weather (number of interviewees in parentheses). Interviewees were asked to outline the key space weather threats they were concerned about, the expected spatial and temporal impacts of these threats, and the different mitigation strategies they currently utilize. Explicit information was gathered regarding the expected impact resulting from different levels of space weather forecasting. We consequently describe a set of evidence‐based scenarios that combine (i) modeled outputs from the vulnerability assessment, (ii) evidence from the U.K. National Risk Register, and where data are unavailable (iii) qualitative information obtained from expert elicitation.

Evidence gathered from the Royal Academy of Engineering report by Cannon et al. (2013), later used for the U.K. National Risk Register, states that thermal heating could damage approximately 13 EHV transformers from a Carrington‐sized event. This is the infrastructure operator's own assessment, and includes two substations experiencing catastrophic damage, leading to disconnection from the transmission grid for potentially two to four months. This estimate is based on the transmission operator's own analysis, with full access to asset vulnerability, existing mitigation, and knowledge of stockpiled transformers. Using this information, we consequently scale the restoration periods for different event sizes and forecasting capabilities via expert elicitation with the U.K. MOSWOC. As we do not explicitly know which nodes are most at risk, we take the average population and employment characteristics of failed nodes, for each scenario, from the simulated vulnerability analysis. Additionally, voltage instability zones are identified using expert elicitation and are corroborated using transformer densities from the developed infrastructure model. Table III provides a description of each scenario by impact type. We assume a linear temporal restoration process for each scenario.

**Table III:** Scenario Description Based on Event Size and Forecasting Capability

Event	Impact Type	Dimension	No Forecast	Current Forecast	Enhanced Forecast
1‐in‐100 year	Voltage collapse	Spatial	National grid collapse	Three voltage instability regions	One voltage instability region
		Temporal	Five days	Two days	One day
	Thermal heating	Spatial	Two nodes	Two nodes	One node
		Temporal	10 weeks (extended offsite transformer replacement)	Six weeks (offsite transformer replacement)	Four weeks (expedient offsite transformer replacement)
1‐in‐30 year	Voltage collapse	Spatial	Two voltage instability regions	One voltage instability region	–
		Temporal	Two days	One day	–
	Thermal heating	Spatial	One node	–	–
		Temporal	Six weeks	–	–
1‐in‐10 year	Voltage collapse	Spatial	One voltage instability region	–	–
		Temporal	12 hours	–	–
	Thermal heating	Spatial	–	–	–
		Temporal	–	–	–

If no forecasting capability is available and multiple substorms are experienced, this dramatically increases the probability of a national voltage collapse. Therefore, we use this as the basis of the 1‐in‐100‐year event if no forecasting capability is available. This situation would necessitate “BlackStart” where the grid must be brought back online via plants capable of using onsite generators, taking up to five days (Cabinet Office, 2017). Damage is also caused to two network nodes, requiring transformer replacement from an offsite location.

If the current forecast is available, interview evidence suggests that mitigation actions for a 1‐in‐100‐year event would cause blackouts in a limited number of voltage instability regions. We assume this takes place in three regions as the AE shifts equatorward, with one very intense substorm affecting Birmingham, and another affecting both the Manchester and Yorkshire regions. Two nodes require transformer replacement from an offsite location taking six weeks to complete. In an enhanced forecast scenario, a 1‐in‐100‐year event may cause only limited short‐term power loss to one voltage instability region such as Birmingham and the West Midlands. Damage from thermal heating could be limited to only a single node, and expedient offsite replacement of transformer assets could be carried out in four weeks. For the 1‐in‐30‐year scenarios, the potential effects are limited, with an enhanced forecast leading to no impacts. Similarly, in the 1‐in‐10‐year scenarios a worst case would involve short‐term blackouts in a single voltage instability region if no forecast was available, otherwise no impacts would take place (as is consistent with our current experience of space weather). We do not test scenarios for events less frequent than 1‐in‐100‐years, due to the increased level of uncertainty.

### 3.7. Direct and Indirect Impacts

Direct impacts are measured by (i) the proportion of the population without power and (ii) local employment disruption by broad industrial group. Voltage instability impacts are calculated by aggregating population and employment within voltage instability regions. For thermal heating risk, we take the average node characteristics from failed nodes by scenario, over 1,000 simulation runs.

Second, we use the Oxford Economics Global Economic Model (OEM) to understand the impact on GDP. This is a widely employed macroeconomic model with users including the International Monetary Fund and World Bank. Multivariate forecasts are produced for many economies, but here we focus only on the United Kingdom. The modeling approach adopts Keynesian principles in the short run, and monetarist principles in the long run. The demand side determines short‐run output, while in the long term supply‐side factors determine output and employment. We quantify the indirect economic impact as 1‐year deviation from baseline growth starting in Q1‐2018, given a demand‐side economic shock due to reduced private consumption from households being without power. Private consumption is affected as consumers are unable to complete daily economic transactions. We parameterize a private consumption shock $cs_{i}$ based on the population disruption from both thermal heating ${\mathrm{PTH}}_{\mathrm{it}}$ and voltage instability ${\mathrm{PVS}}_{\mathrm{it}}$ at time *t* in the *i*th scenario as follows:

(3) $$cs_{i}=\frac{\left({\mathrm{PTH}}_{\mathrm{it}}+{\mathrm{PVS}}_{\mathrm{it}}\right)}{P\;\cdot \left(\frac{w}{q}\right)},$$

where *P* is the total population (63.3 million), *w* is the number of working days per year (280), and *q* is the number of quarterly periods per year. This process is repeated for a quarterly supply‐side labor shock $ls_{i}$ to represent reduced labor supply, as employees are unable to travel to work or log in remotely. The summation of labor disruption from both thermal heating ${\mathrm{LTH}}_{\mathrm{it}}$ and voltage instability ${\mathrm{LVS}}_{\mathrm{it}}$ at time *t* in the *i*th scenario is as follows:

(4) $$\;ls_{i}=\frac{\left({\mathrm{LTH}}_{\mathrm{it}}+{\mathrm{LVS}}_{\mathrm{it}}\right)}{L\;\cdot \left(\frac{w}{q}\right)},$$

where the total labor force is represented by *L* (30.9 million), *w* is the number of working days per year (280), and *q* is the number of quarterly periods per year. The analysis focuses on the short‐term GDP impacts, and not on the long‐term equilibrium position of the economy. Finally, the model output for each scenario is subtracted from the baseline growth estimate, to obtain the GDP impact per scenario. The Global Economic Model utilized is available from Oxford Economics, obtained here under academic license, and therefore is accessible for other analysts wishing to reproduce similar analyses. The model partially solves rescheduling and input substation effects over a 1‐year period, but a longer‐term analysis would more effectively quantify any rebound effects associated with postdisaster recovery.

## 4 RESULTS

### 4.1. Magnetospheric Substorm Probability

Following the method outlined in Section 3.1, we construct time sequences of substorms to quantify the uncertainty associated with the rotation of the Earth, and now report the magnetospheric substorm probability for each of the risk scenarios in Table IV. This shows how the likelihood of a very intense substorm over the United Kingdom changes between different event sizes. For a 1‐in‐10‐year event there is only an 8% probability of being affected by a single substorm, although this rises to 17% for a 1‐in‐30‐year event. In these circumstances we would not expect to see more than a single substorm taking place, for which there is a very low probability.

**Table IV:** Estimated Likelihood of Very Intense Substorms Over the United Kingdom

	Number of Very Intense Substorms Over the United Kingdom			
Scenario	0	1	2	Total Cases
1‐in‐10 year	22 (92%)	2 (8%)	0 (0%)	24
1‐in‐30 year	20 (83%)	4 (17%)	0 (0%)	24
1‐in‐100 year	7 (29%)	12 (50%)	5 (21%)	24

However, for a 1‐in‐100‐year event the probability of being affected increases significantly. For example, there is a 50% probability that the United Kingdom would experience a very intense substorm, and a 21% probability of two very intense substorms, taking place across the nation's geographic area.

### 4.2. GIC Vulnerability Assessment

**Figure 3:**
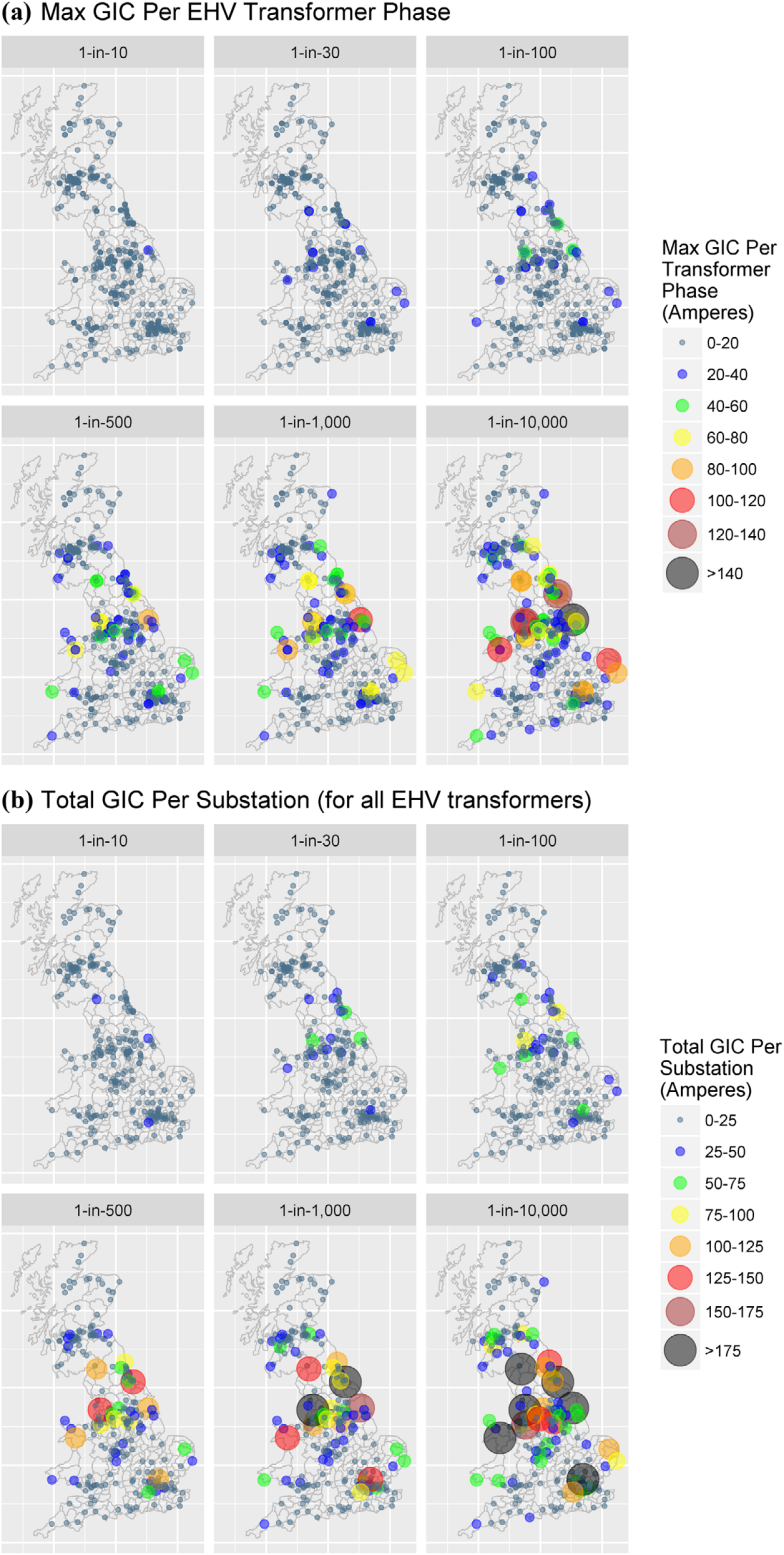
(a) Max GIC per EHV transformer phase and (b) total GIC per substation.

We find that the peak GIC per transformer phase (using one‐minute sampled data) ranges from a median of 2 amperes (A) and maximum of 20 A in the 1‐in‐10‐year scenario, to a median of 11 A and a maximum of 156 A in the most extreme 1‐in‐10,000‐year scenario. The maximum GIC experienced per transformer phase is illustrated in Fig. 3(a), showing some of the largest asset exposures are in the northeast and northwest of England. Supporting Information Appendix D provides detailed simulation summary statistics.

Fig. 3(b) illustrates the total GIC per node based on the EHV transformers present. The exposure was minimal for a 1‐in‐10‐year event with a median of 2 A and a maximum of 29 A, whereas in the most extreme event the median was 11 A with a maximum of 245 A. The difference between the median and the maximum exposure indicates large GICs flow in particular “hot spots” at the eastern and western edges of the network. An interesting finding is that the magnitude of exposure is different between the GIC per transformer phase and total GIC per node (the former being of greater importance).

**Figure 4:**
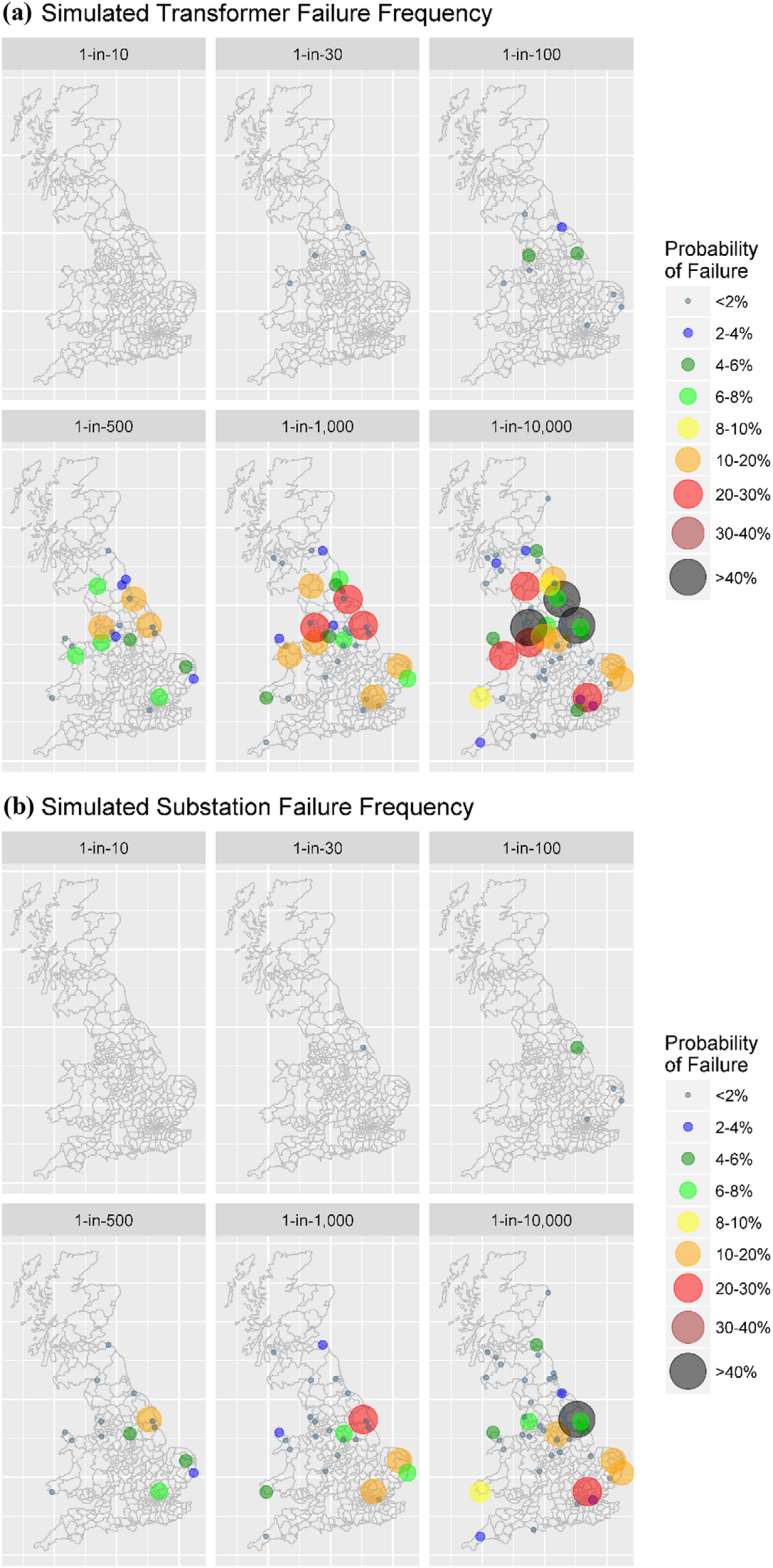
(a) Simulated transformer failure frequency and (b) simulated substation failure frequency.

The frequency of failures based on the random allocation of unknown transformer characteristics is illustrated in Fig. 4(a). The northeast and northwest of England had several transformers with a high frequency of failure, along with East Anglia and Wales. This is consistent with impacts reported during the 1989 storm, with transformer failure at Norwich (East Anglia) and significant transformer noise at Pembroke, southwest Wales (Smith, 1990). No transformer damage takes place from a 1‐in‐10‐year event, along with minimal impacts from a 1‐in‐30‐year event. For the most probable extreme event, the 1‐in‐100‐year scenario produced a transformer failure probability in at‐risk nodes up to 5%. This increased to over 50% in the most extreme 1‐in‐10,000‐year event.

However, a single transformer failure may not lead to the loss of the whole node. Consequently, we visualize the simulation results for the frequency of substation failure in Fig. 4(b). Under these simulation conditions, it illustrates there is a small probability of power loss due to thermal heating, with the most vulnerable nodes generally positioned at the east and west coastal edges of the network. This probability ranges from 4% for the 1‐in‐100‐year event up to over 40% for a 1‐in‐10,000‐year event, with these effects at the eastern and western edges of the network, particularly at Sizewell, Norwich, and Pembroke.

### 4.3. Socioeconomic Impact Results

The direct impacts in the no forecast scenarios were significantly higher when compared to other outcomes. In a 1‐in‐100‐year event with no forecast, initial disruption affected over 60 million people and almost 30 million workers. This impact is substantially reduced under the current forecast capability where direct population disruption dropped to 13 million and employment disruption dropped to 6 million. Enhanced forecasting capability reduced both population and labor disruption to a minimal level, particularly in smaller, more frequent events.

Table V details the level of population and labor disruption on day 1 of each scenario, as well as the consequential quarterly shock sizes applied to consumption and labor in the OEM macroeconomic model. We find that in a 1‐in‐100‐year event with no forecast, the GDP impact reached approximately £15.9 billion, with this dropping to £2.9 billion based on the current forecast capability, and £0.9 billion with an enhanced forecast. For no forecast, in a 1‐in‐30‐year event the GDP impact was £1.9 billion, decreasing to £0.4 billion under current forecast. Finally, for a 1‐in‐10‐year event with no forecast the impact was £0.4 billion.

**Table V:** Economic Impact by Scenario

Event	Forecast Capability	Total Population Disruption (Millions) (Day 1)	Total Labor Disruption (Millions) (Day 1)	OEM Shock Type	OEM Shock	GDP Loss (Billions, GBP)
1‐in‐100 year	No Forecast	62.1	29.4	Consumption	0.9518	15.9
				Labor	0.9527	
	Current Forecast	19.4	8.8	Consumption	0.9911	2.9
				Labor	0.9913	
	Enhanced Forecast	8.1	3.7	Consumption	0.9974	0.9
				Labor	0.9974	
1‐in‐30 year	No Forecast	13.3	6.1	Consumption	0.9942	1.9
				Labor	0.9944	
	Current Forecast	5.2	2.4	Consumption	0.9987	0.4
				Labor	0.9988	
	Enhanced Forecast	–	–	Consumption	–	–
				Labor	–	
1‐in‐10 year	No Forecast	5.2	1.4	Consumption	0.9987	0.4
				Labor	0.9993	
	Current Forecast	–	–	Consumption	–	–
				Labor	–	
	Enhanced Forecast	–	–	Consumption	–	–
				Labor	–	

## 5. DISCUSSION

Estimating the potential socioeconomic impacts of space weather is a challenge as many areas of uncertainty exist, both in our current scientific and engineering understanding of this threat, and in current data and modeling methodologies. In this discussion we examine the findings of the analysis in relation to the research questions. Supporting Information Appendix E discusses the uncertainty associated with the data and modeling approaches utilized, and areas for future research.

### 5.1. What Is the Probability of CNI Being Affected by Intense Magnetospheric Substorms?

Time sequences of substorms were constructed to estimate probabilities of geographic impact under different 1‐in‐10‐year, 1‐in‐30‐year, and 1‐in‐100‐year levels. Over a full 24‐hour Earth rotation, the risk at any particular location is modest for the 1‐in‐10‐year and 1‐in‐30‐year events, but escalates markedly when we move to a 1‐in‐100‐year event. Hence, we find that the United Kingdom was unlucky to experience the very intense substorm that caused two transformer failures during the 1989 event (the basis of the 1‐in‐30‐year scenario) as the likelihood of geographic impact was only 17%, so it was a relatively rare but not improbable occurrence. Moreover, the results suggest it was entirely reasonable that the United Kingdom experienced no substorm, and consequently no power grid problems during the 2003 event (the basis of the 1‐in‐10‐year scenario), as the substorm probability was only 8%.

Finally, we find that a Carrington‐class event (the 1‐in‐100‐year scenario) has a very high probability (71%) of producing very intense substorms over the United Kingdom that could disrupt the power grid, resulting from a 50% likelihood of a single very intense substorm and a 21% likelihood of two very intense substorms. In this latter case, the second event could occur many hours (perhaps 24 hours) after the initial event, thus posing a serious challenge to recovery efforts. During expert elicitation interviews it was expressed that two very intense substorms, particularly with no forecast available, would dramatically increase the probability of significant power grid difficulties, increasing the likelihood of a national grid collapse.

### 5.2. How Vulnerable Are Specific Electrical Transmission CNI Assets and Nodes to GIC Exposure?

As detailed data on transformer design characteristics are unavailable, we explored the sensitivity of transformer and node failure based on the random allocation of this parameter. Moreover, as there is little agreement regarding extreme events, we explore the sensitivity of the results to increasingly large geomagnetic storms.

Under the simulation conditions tested, the probability of transformer failure from thermal heating was generally quite low for more frequent events, but increased considerably in the more extreme scenarios, where the failure rate for some assets exceeded 50%. This translated to relatively modest impacts when evaluating the probability of node failure, as it would take more than half of the available EHV assets to fail for a blackout to be caused by the loss of a network node. Consequently, no nodes failed in the smaller, more frequent storm scenarios, but the failure probability ranged between 2% and 40% in the more extreme events. However, the actual failure rate depends on the asset management practices of the infrastructure operator, as the random allocation of transformer types introduces uncertainty in these results. For example, the model may overestimate the vulnerability of urban locations that are likely to have been the focus of previous resilience efforts, while also underestimating the vulnerability of more rural substations. Either way, the results of this analysis provide evidence supporting grid configuration policies to place newer, more GIC‐resistant designs at substations that contain transformers with older, less GIC‐resistant designs. Finally, while the scenarios tested here have emphasized impacts at higher latitudes within the United Kingdom, such as the northeast and northwest of England, we must also avoid complacency about impacts in the south.

### 5.3. What Are the Potential Socioeconomic Impacts of Electrical Transmission CNI Failure Due to Space Weather, Under Different Forecasting Capabilities?

Space weather forecasting is a recognized mitigation for managing the risk posed by space weather, and CNI operators are dependent on a forecast being available to take operational decisions to reduce exposure. The results were most concerning for the no forecast scenario, where the GDP impact reached almost £16 billion in the largest event. Given that space weather forecasting uses data from a limited number of satellites, some of which are nearing the end of the expected lifespan, this is concerning. Many existing satellites are research missions (hence, effectively nonoperational), and while high‐quality data are collected, transmission to Earth may not take place in an optimal timeframe to support operational space weather forecasting.

The status quo in terms of forecasting capability is unlikely to be maintained. Limited, or no, investment will see capability decline from today's skill levels, increasing the risk of CNI failure and consequential economic loss. Investment in the relevant space‐borne monitoring is expected to lead to operationally reliable data streams that would achieve the enhanced capability described in Supporting Information Appendix C. Without this investment economic losses would be expected to be greater and fall somewhere between the current and no forecast capabilities. Based on the analysis presented here, there is evidence to support investment in maintaining forecasting capabilities, as well as predictive models and risk communication, as they provide early warning for the low‐probability, high‐impact threats caused by space weather. Importantly, the reduced economic impacts associated with better space weather forecasting capabilities depend on utilities having effective operational mitigation plans. While this is the case for the U.K. national grid, it might not apply in other regions where application of this risk framework may take place.

## 6. CONCLUSION

The time‐shift analysis of the geomagnetic storm catalogue suggests that U.K. risk is modest for the 1‐in‐10‐year and 1‐in‐30‐year levels, but significantly increases for a 1‐in‐100‐year event. Moreover, in a sensitivity analysis of the vulnerability of transformer assets, we find the failure probability ranges from below 2% for minor events, to 4% for a Carrington‐sized event approximately 1.4× larger than the 1989 event. The probability of substation failure ranged from negligible in smaller events, to over 40% based on the theoretical upper limit proposed by Vasyliūnas (2011).

We find that for a Carrington‐sized 1‐in‐100‐year event with no space weather forecasting capability, the GDP loss to the United Kingdom could be as high as £15.9 billion, with this figure dropping to £2.9 billion based on current forecasting capability. However, with existing satellites nearing the end of their life, current forecasting capability will decrease in coming years. Therefore, if no further investment takes place critical infrastructure will become more vulnerable to space weather. Additional investment could provide enhanced forecasting, reducing the economic loss for a Carrington‐sized 1‐in‐100‐year event to £0.9 billion.

Partial information often prevents comprehensive risk assessment. The contribution of this article is to provide a general framework for the risk assessment of the socioeconomic impacts of space weather for the United Kingdom. Applying this to the British high‐voltage network forms one of the first socioeconomic assessments undertaken for this threat (however, application to other countries would require further adaptation). Unlike other analyses undertaken hitherto, we properly address the general geophysical risk, asset vulnerability, and CNI network structure. This has required a multidisciplinary approach, utilizing methods from space physics, geophysics, electrical engineering, and economics, but provides a step toward the standardization of space weather risk assessment. Importantly, we were conservative in our treatment of the space and geophysical hazard, estimating potential minimum impacts.

Further research must enhance this simulation to encompass the relationship between GIC, reactive power demand, and the available capacity (and critical paths) of spinning reserve under different forecasting capabilities. Future analyses should attempt to quantify the impact (and financial cost) of infrastructure “hardening” via retrofitting. Finally, to capture the true socioeconomic impacts of space weather, disruption in other interdependent infrastructure systems must also be quantified, potentially increasing the economic impacts presented here.

## Supporting information


**APPENDIX A**: DETAILED METHODOLOGY ON DEFINING THE SPACE THREAT
**Fig. A1**. Variation of the auroral electrojet index AE during the great geomagnetic storm of March 13–14, 1989, annotated to show occurrence of major GIC impacts.
**Fig. A2**. BGS2012 Conductance Model 2D map for the top 3 km of the crust.
**Fig. A3**. Lagrange positions.
**Table AI**. Substorm Scenario Derived from the 1989 Scenario; Day 1 Matches March 13
**Table AII**. 1‐in‐100‐Year Substorm Scenario; Day 1 Matches September 2
**APPENDIX B**: DETAILED METHODOLOGY ON GIC ESTIMATION
**APPENDIX C**: OVERVIEW OF SPACE WEATHER FORECASTING CAPABILITIES
**APPENDIX D**: MODEL SUMMARY STATISTICS
**APPENDIX E**: KEY DATA AND MODELING UNCERTAINTIES
