# Supplementary material for: A Risk Assessment Framework for the Socioeconomic Impacts of Electricity Transmission Infrastructure Failure Due to Space Weather: An Application to the United Kingdom
Source: Risk Anal. 2018 Nov 8;39(5):1022–43. doi: 10.1111/risa.13229 (PMC6936226; doi:10.1111/risa.13229)
Supplement: Supplementary file 1 — APPENDIX A: DETAILED METHODOLOGY ON DEFINING THE SPACE THREAT Fig. A1. Variation of the auroral electrojet index AE during the great geomagnetic storm of March 13–14, 1989, annotated to show occurrence of major GIC impacts. Fig. A2. BGS2012 Conductance Model 2D map for the top 3 km of the crust. Fig. A3. Lagrange positions. Table AI. Substorm Scenario Derived from the 1989 Scenario; Day 1 Matches March 13 Table AII. 1‐in‐100‐Year Substorm Scenario; Day 1 Matches September 2 APPENDIX B: DETAILED METHODOLOGY ON GIC ESTIMATION APPENDIX C: OVERVIEW OF SPACE WEATHER FORECASTING CAPABILITIES APPENDIX D: MODEL SUMMARY STATISTICS APPENDIX E: KEY DATA AND MODELING UNCERTAINTIES [file RISA-39-1022-s001.docx]

Appendix A Detailed methodology on defining the space threat

This appendix outlines how we have developed simple scenarios to explore the impact of space weather on power grids in countries at high and mid-latitudes, regions where the strongest GICs are thought to be associated with magnetospheric substorms (Ngwira et al. 2015; Pulkkinen, Bernabeu, Eichner, Viljanen and Ngwira, 2015). These substorms are a fundamental dynamical cycle of Earth’s magnetosphere, in which energy is extracted from the solar wind, stored in the tail of the magnetosphere and then explosively released (Dungey, 1961), each cycle typically lasting one to two hours, repeating as long as there is a supply of energy from the solar wind. Much of the explosive energy release is directed to Earth where it can produce a burst of intense aurora and electric currents in the upper atmosphere, leading to large geomagnetic variations which can spread from high to mid-latitudes during intense events. A geomagnetic storm typically contains a series of substorms and thus has the potential to produce a series of bursts of GIC in power grids.

Substorms are typically characterised using the auroral electrojet indices, a set of indices derived from geomagnetic measurements at 13 observatories in the northern hemisphere auroral zone, which monitor the electric currents (electrojets) flowing in Earth’s ionosphere at auroral latitudes (Davis & Sugiura, 1966). Therefore, we have built our scenarios using values of these indices for two of the largest space weather events in recent decades, namely the geomagnetic storms of March 1989 and October 2003. We specifically focus on the AE index which represents the overall activity of the electrojets. Other indices such as AU and AL represent the strongest eastward and westward currents in the electrojets and are of interest for future studies, but in this first study we focus on AE.

Fig. A1 shows AE data for the March 1989 storm. The raw AE data is quite spikey as shown by the grey trace in the background. To highlight substorms, rather than short-lived features, we have smoothed these data with a 31-min running median (time-tagged to the central data point in each median). The use of a 31-minute window highlights variation in substorm timescales of 1-2 hours and use of a median ensures a focus on general trends that is not influenced by isolated extreme values. The smoothed AE for March 1989 is shown by the blue trace in Fig. A1. The main storm started at 01:27 UTC on 13 March. The event is marked by a vertical green line and indicates the arrival at Earth of a major CME, one whose impact significantly compressed the geomagnetic field, a compression observed by ground-based magnetometers across the world (Observatori de l‘Ebre, 2018). Following the onset of the main storm AE showed high activity throughout the whole of 13 March and the morning of 14 March. The main storm was preceded by significant activity in AE late on both 12 and 14 March, reflecting that the main storm was the central part of a two-week period of intense space weather (Allen, Sauer, Frank and Reiff, 1989).

The high peaks in this AE sequence indicate substorms that had major space weather effects. For example, the peak during the morning of 13 March is associated with the voltage collapse of the Hydro-Québec power grid in Canada (Bolduc, 2002), whilst the peak during the evening of 14 March was associated with a spectacular auroral display over southern England. To extract a simple scenario from this sequence of AE peaks we need to do two things: (a) set a threshold that we use to select the most intense substorms, and (b) associate the geomagnetic footprints of those substorms with particular geographic regions (such that power grids in those regions will be at risk from GIC driven by the substorm).

For the threshold we have set this at AE > 1900 nT, giving us a total of three very intense substorms during the main storm, indicated in red in Fig. A1. This is a fairly arbitrary choice of threshold but does match a number of major space weather impacts and effects reported as noted in Fig. A1. Most obviously the Hydro-Québec voltage collapse on the morning of 13 March, as well as the tripping of two UK transformers as reported by Smith (1990) and Erinmez, Kappenman and Radasky (2002). An internal technical report on the 1989 storm by the then nationalised electricity generator shows that two transformers, one at Norwich in East Anglia and one at Indian Queens in Cornwall, tripped out during the very intense substorm early on 14 March. The Norwich transformer also tripped out during a substorm on the evening of 13 March, one peaking just below our 1900 nT threshold. This suggests that this threshold is conservative, and thus appropriate for our aim of not overstating the risk.

Fig. A1. Variation of the auroral electrojet index AE during the great geomagnetic storm of 13/14 March 1989, annotated to show occurrence of major GIC impacts.


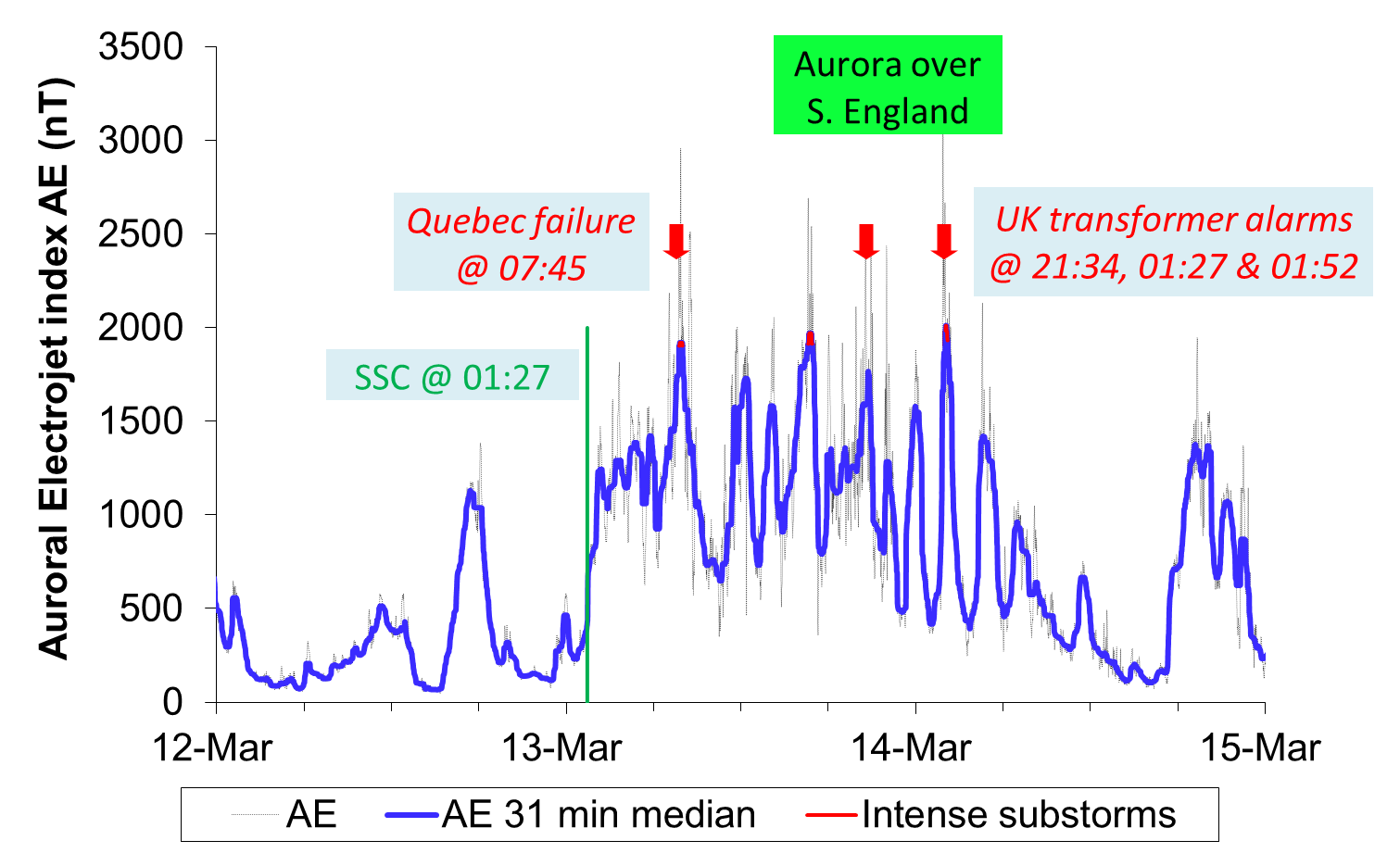


For the footprints we have set this at the region where local time near 02:30 at the time of the substorm. This was originally chosen to match the timing of the Hydro-Québec voltage collapse around 03:00 local time and the tripping of the two UK transformers, which was thought to have occurred during an intense substorm over southern England peaking around 02:00 local time. The latter speculation is now supported by recent access to the above report on UK power impacts, which confirms that the two transformers tripped between 01:20 and 02:00 local time. As noted above, that report also points to an additional trip during an earlier substorm, suggesting that a wider local window should be considered in future work. However, for the present work we focus the scenarios on a limited time window, which makes these scenarios fairly conservative in the assessment of space weather impacts on power grids. A wider window will require a more nuanced approach, e.g. a graduated weighting of impact by local time, for which we currently lack data.

This combination of AE thresholds and local time footprints allows us to build scenarios that are simply time sequences of intense substorms, each of which has a footprint in a particular time zone. Table AI below shows the scenario extracted from the AE data for the 1989 storm. As discussed in the main paper we use this as our 1-in-30-year scenario, and also use a similar scenario derived from the October 2003 storm as our 1-in-10 year scenario.

Table AI. Substorm scenario derived from the 1989 scenario. Day 1 matches 13 March.

| *Day* | *UTC at peak* | *Region at 02:30 local time* |
| --- | --- | --- |
| 1 | 07:55 | Eastern Canada, North East US |
| 1 | 16:45 | Japan, SW Australia |
| 2 | 02:05 | Western Europe, including UK |

To scale up to a 1-in-100-year scenario, we adapt detailed data from the March 1989 storm guided by the limited data from the storm of August/September 1859. This is widely considered as a 1-in-100-year space weather event, e.g. as in the collection of papers edited by Clauer & Siscoe (2006). Our knowledge of the 1859 event includes: (a) that it was a pair of geomagnetic storms, one large followed by an even larger event, (b) accurate times of the SSC for each storm (Stewart, 1861), and (c) an estimate of the Dst geomagnetic index for the larger storm (Siscoe et al. 2006). We use this knowledge to construct a representative AE timeline for the 1859 storm using two copies of the 1989 AE timeline. We time-shift each copy so that the Universal Time and day-of-year of the SSCs match those reported in Stewart (1861). We also increase the AE values in the second copy by a factor that reflects the greater strength of that storm. We derive that factor using estimates of the Dst index for the 1859 and 1989 storms (the AE index is available only from 1957). Although Dst is a measure of the storm severity, it is not a good measure of the auroral current systems. However, Dst is a good measure of the ring current. Since the repeated injection of particles towards the Earth during substorms contributes to the ring current, and in the absence of any better measurement, we have scaled the AE time series by the ratio of the Dst index between 1989 and 1859. We then use the resulting AE timeline to derive a 1-in-100-year scenario, which is shown in Table AII below.

Table AII 1-in-100-year substorm scenario. Day 1 matches 2 September

| *Day* | *UTC at peak* | *Region at 02:30 local time* |
| --- | --- | --- |
| -3 | 04:54 | Atlantic Ocean |
| 1 | 08:16 | Eastern US and Canada |
| 1 | 09:18 | Central Canada and US |
| 1 | 11:24 | Pacific Ocean |
| 1 | 15:05 | New Zealand |
| 1 | 15:52 | Pacific Ocean |
| 1 | 17:38 | SE Australia, Japan |
| 1 | 20:17 | Central Siberia, Western China |
| 2 | 00:16 | European Russia |
| 2 | 03:30 | Atlantic Ocean |
| 2 | 05:36 | Atlantic Ocean |
| 2 | 08:09 | Eastern US and Canada |
| 2 | 23:36 | European Russia |
| 3 | 12:47 | Pacific Ocean, Alaska |
| 6 | 18:28 | Eastern China |
| 7 | 17:11 | SE Australia, Japan |

We must emphasise that, in building this scenario, we have smoothed the AE index by a 31-minute running median so as to highlight substorms that re-occur on a timescale of a few hours Borovsky & Yakymenko (2017). However, we recognise that ionospheric currents that affect the power grid may occur on much shorter timescales and if we had used a shorter running median we would obtain a smoothed AE index with higher peaks (e.g. reducing the smoothing window size to 15 minutes increases peaks by 10 to 15%). Scaling by the Dst index could then imply greater disruption. At this stage of our research the link between AE and power grid effects is uncertain and therefore we have kept to our more conservative approach but note that the disruption could be even higher.

One striking thing about the 1-in-100-year scenario is that whilst the affected regions are spread around the world, there is only a very limited impact on Western Europe, including the UK. This is largely a consequence of the SSC time for the larger storm (05:00 on Day 1 of the 1-in-100-year scenario). To obtain a more realistic assessment of the 1-in-100-year impact on the UK we must consider a range of SSC times covering a full 24 hours of Universal Time, equivalent to a large CME arriving at any time of day. We do this quite simply by creating 24 instances of the scenario, with the SSC time stepped forward by 1 hour from one scenario to the next. We then analyse this ensemble of 24 instances to find how many scenarios give 0, 1 or more intense substorms over the UK with the results shown in the last row of Table A3 below. For comparison, we also apply this time-shift method to the 1-in-10 and 1-in-30-year scenarios.

Appendix B Detailed methodology on GIC estimation

We use a three-step process for computing GIC in EHV transmission infrastructure. The first step involves the measurement of the rapidly varying geomagnetic field using ground-based observatories. The second step is the computation of a map of the induced geoelectric field using either plane-wave or thin-sheet approximation (in which we use the latter here) from the interaction of the magnetic field with a ground conductivity model. The thin-sheet method has been validated against geoelectric field measurements in the UK and compares well with 3D conductivity models at periods longer than 1 minute. Finally, we utilise the topology, location and resistance characteristics of the electricity transmission infrastructure network model articulated in the previous section. This is placed onto the geoelectric field map to deduce the GIC (Beggan et al. 2013).

The spatial variation of the magnetic field was estimated using minute-mean data for the 1989 (as the 1-in-30-year exemplar and up-scaled by Dst for rarer events) and 2003 (1-in-10-year exemplar) storms, interpolated over a large region using the Spherical Elementary Current Systems method (Amm & Viljanen, 1999), as described in detail in McLay & Beggan (2010). The interaction of this rapidly varying magnetic field with the conductive Earth was then computed using the thin-sheet method (Vasseur & Weidelt, 1977). This determined the surface electric field arising at a particular frequency from layers of conductive material in the subsurface. The chosen frequency (or period) of the rate-of-change of the magnetic field is related to its penetration depth, which in this study is 600 seconds; a value that has been validated against measured electric field and GIC data where short-period local fluctuations are removed to leave the regional scale data (McKay, 2004).

The UK 2D surface conductance model is derived from the analysis of the conductivity properties of bedrock, based on the British Geological Survey 1:625,000 geological map of Great Britain. The model, described by Beamish & White (2012), uses information obtained from airborne geophysical surveys across the UK to determine the conductance to a depth of 3 km for the thin-sheet part of the model. For the offshore regions, the bathymetry and a uniform value of sea water conductivity (4 S/m) are used to determine conductivity. Fig. A2 illustrates the BGS2012 Conductance Model. At depth, below the thin-sheet model, a 1D model of resistivity down to 1000 km is used, based on information from magnetotelluric studies of the UK (e.g. McKay, 2004). A national geophysical survey analogous to the US ‘EarthScope’ project is not available for the UK, though a current project to improve geophysical knowledge of the UK’s conductivity structure is underway ([SWIGS](http://www.geomag.bgs.ac.uk/research/SWIGS/home.html)).

Fig. A2 BGS2012 Conductance Model 2D map for the top 3km of the crust


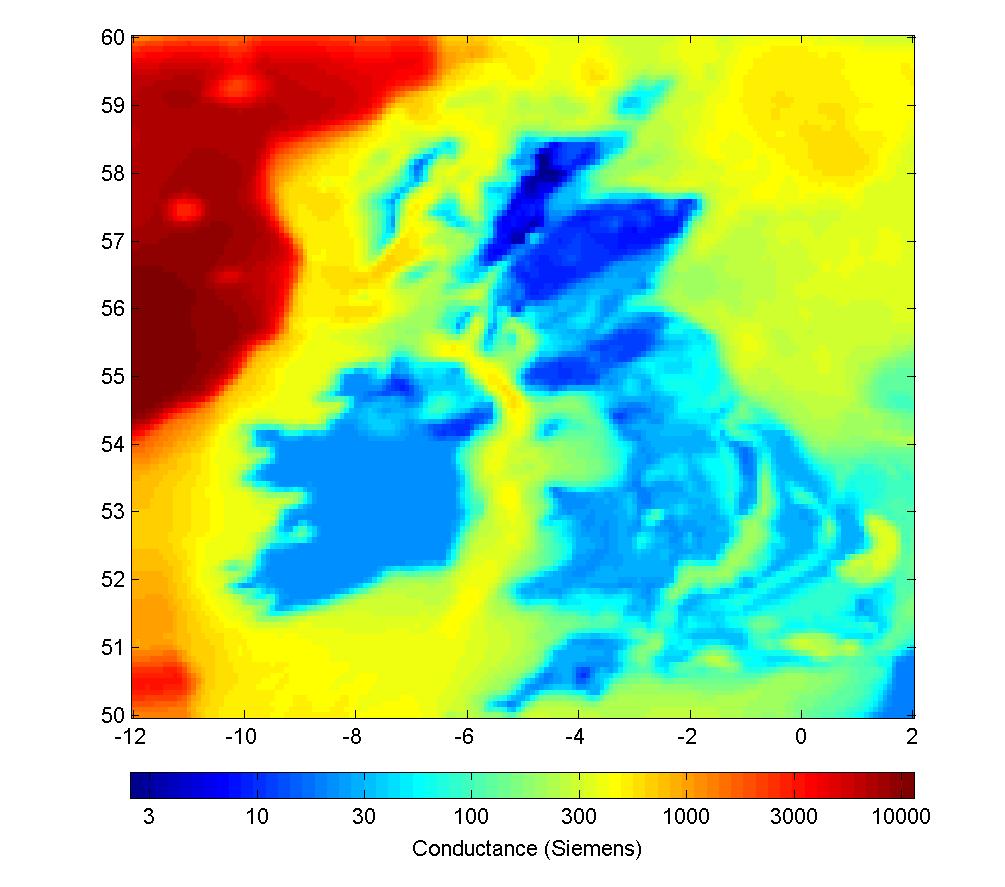


Appendix C Overview of space weather forecasting capabilities

Space weather forecasting can provide advanced warning to CNI operators that a solar storm has taken place on the surface of the Sun, and specific solar phenomena such as a CME may be directed at Earth. Data are used from a limited number of satellites, some of which are nearing the end of the expected lifespan, for example, some research satellites are already not considered operational. Whilst these research satellites collect high quality data, they may not be downloaded to Earth in an optimal timeframe to support operational space weather forecasting. As the implementation of operational mitigations by CNI operators has a substantial monetary cost associated, the confidence in the available forecast is an important variable.

The current level of capability depends on a mix of research and operational satellites which include DSCOVR, SOHO, and Advanced Composition Explorer (ACE) orbiting the Sun with the Earth. They are in direct line between the Sun and the Earth near the Lagrange 1 (L1) point where the combined gravitational attraction of the Sun and Earth allows quasi-stable satellite orbits. Quasi-stable orbits also exist at four other Lagrange point (L2 to L5) related to the Sun and Earth as shown in Fig. A3. The Geostationary Operational Environmental Satellites (GOES) exist along with SDO in near-Earth orbit and additionally STEREO A which is trailing the Earth, currently in a 1AU solar orbit at approximately 120° (halfway between L5 and L3).

Fig. A3 Lagrange positions


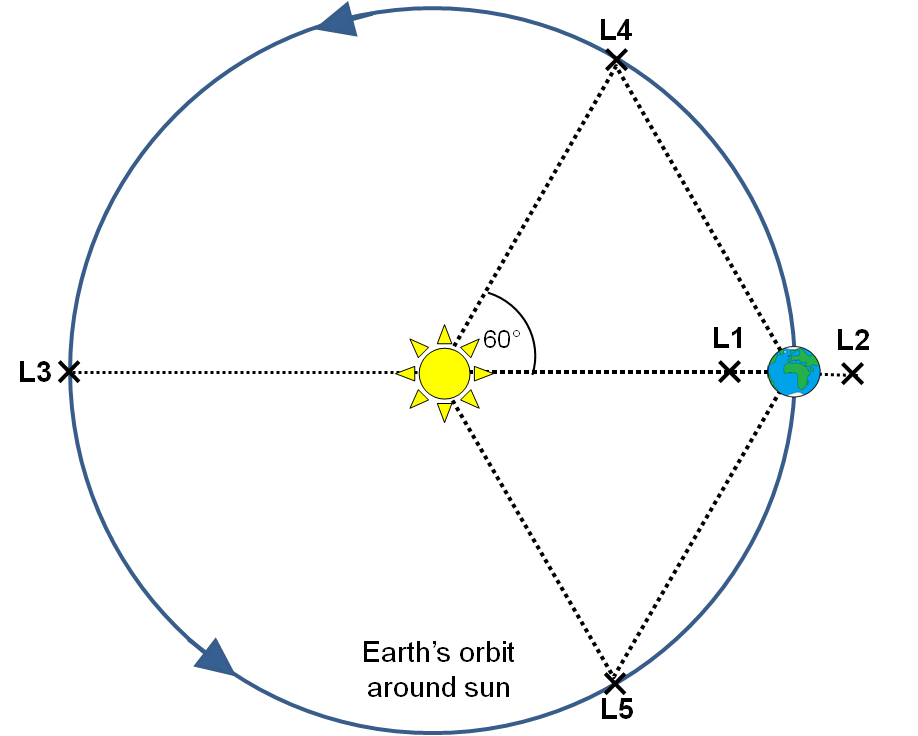


Current forecast capability allows complex sunspot regions to be identified 3-4 days before the Earth is in the direct line of any CMEs the region may produce. Any CME would then be detected by imagers on SOHO and STEREO A shortly after launch and the magnetic polarity of the CME 15-20 minutes (for a very fast CME) warning from in-situ measurements by DSCOVR/ACE at L1 point.

An enhanced level of forecasting would reflect the launching of an additional L5 satellite mission alongside a replacement L1 mission both providing operationally reliable data streams. The L5 mission would increase the lead time for identifying and monitoring complex active regions by a further 3 to 4 days and would provide a permanent side-on view of the interplanetary space between the Sun and Earth. This capability would improve the level of reliability and hence confidence in space weather forecasts, increasing the likelihood that infrastructure operators would take the appropriate operational mitigations when a threat is posed.

Appendix D Model summary statistics

| **Scenario** | **Type** | **Minimum (Amps)** | **Lower quartile (Amps)** | **Median (Amps)** | **Upper quartile (Amps)** | **Maximum (Amps)** | **Mean (Amps)** | **Standard deviation (Amps)** |
| --- | --- | --- | --- | --- | --- | --- | --- | --- |
| **1-in-10** | **Transformer** | 0 | 1 | 2 | 4 | 20 | 3 | 3 |
| **1-in-30** | **Transformer** | 0 | 1 | 3 | 5 | 37 | 4 | 5 |
| **1-in-100** | **Transformer** | 0 | 1 | 4 | 7 | 52 | 6 | 8 |
| **1-in-500** | **Transformer** | 0 | 2 | 6 | 13 | 89 | 11 | 13 |
| **1-in-1,000** | **Transformer** | 0 | 3 | 8 | 16 | 115 | 14 | 17 |
| **1-in-10,000** | **Transformer** | 0 | 4 | 11 | 22 | 156 | 19 | 23 |
| **1-in-10** | **Node** | 0 | 1 | 2 | 4 | 29 | 4 | 5 |
| **1-in-30** | **Node** | 0 | 1 | 3 | 7 | 58 | 6 | 9 |
| **1-in-100** | **Node** | 0 | 1 | 4 | 9 | 82 | 9 | 13 |
| **1-in-500** | **Node** | 0 | 2 | 6 | 16 | 140 | 15 | 23 |
| **1-in-1,000** | **Node** | 0 | 3 | 8 | 20 | 181 | 19 | 29 |
| **1-in-10,000** | **Node** | 0 | 4 | 11 | 28 | 245 | 26 | 40 |

Appendix E Key data and modelling uncertainties

Space Threat

The key limitation relates to whether the past is a good predictor of the future, especially as we have been conservative in our estimates. For example, these probabilities are based on the analysis of the historical storm catalogue, but we know that we regularly see events which considerably exceed expected maximum values, with a recent example being the Fukushima nuclear power plant disaster in Japan. In this event, a tsunami wave exceeding 13 meters breached the plant’s sea wall defences and led to one of the worst nuclear disasters in history. The tsunami considerably exceeded the maximum expected wave height. This was seen in advance of the disaster as a highly improbable event based on historical data (>1-in-10,000-year), hence why defences had not been engineered to withstand a tsunami of this magnitude. Future research needs to extend the analysis to encompass AE time-series values for the large events prior to 1989, such as 1959, 1960 and 1967.

Network Structure

The electricity transmission infrastructure network model used is a simplification of the actual network, along with the strict definition of node ‘failure’. Modelling the lower voltage electricity distribution network was beyond the scope of this analysis, but doing so would capture the fact distribution nodes (e.g. 132 kV) sometimes have multiple connections to the transmission grid, increasing their level of resilience. Further research should consider using a DC power flow model for the transmission grid, providing more realistic representation of nodes that serve major power plants (e.g. Sizewell), as the loss of these nodes could have much wider system impacts.

Ground threat risk manifestation

When estimating ground conductivity in the UK, the existing state-of-the-art does not yet include 3D ground conductivity modelling. Although data collection efforts are now underway, it is likely to be a few years before enough data are collected to enable this endeavour to begin. In terms of objective validation, the BGS2012 model utilised a thin-sheet approximation that has been verified against measurements of the electric field at Lerwick, Eskdalemuir and Hartland observatories. The analysis presented here is consistent with (Beggan, 2015) whereby the sensitivity of GIC modelling to variable ground conductivity was explored, in which the conclusion was that ground conductivity is a second-order effect and is only significant in around 10% of nodes.

Vulnerability assessment

A limitation of the vulnerability assessment undertaken is that it fails to address reactive power demand and voltage instability risk with the same level of rigour as thermal heating risk. Hence further research needs to attempt to scale the probability of voltage instability based on the reactive power demand in each system area.

Moreover, obtaining further geographical information from National Grid regarding the transformer design characteristics between urban and rural areas could help to refine the vulnerability assessment. In this analysis transformer designs were allocated randomly, however it is likely that assets with lower GIC thresholds exist at the rural edges of the network, where asset replacement is a slower process due to the limited number of customers served per node. In this analysis, the simulation results per node mean we end up with approximately 250,000 people per affected substation on average, which is higher than the 100,000 stated in the National Risk Register. This weakness results from using publicly available data, but further refinement of the simulation conditions could help to address this.

Resilience measures

The current approach to estimating the level of resilience gained from space weather forecasting is relatively qualitative, therefore further analysis should explore the potential use of event trees for evaluating the benefits of different levels of early warning. This type of approach would enable the event stages in different scenarios to be identified, and the probabilities of different event paths to be parameterised by expert elicitation methods. Such an approach would better address the cost-benefit trade-off CNI operators face when dealing with low confidence levels in space weather forecasts.

Scenario specification

Much of the analysis that already exists, particularly in the grey literature, provides little transparent evidence for how key scenario parameters have been determined. Often the descriptions of the expected scenarios have been very qualitative and derived from expert elicitation as we lack evidence to help parameterise more extreme events. Moving towards improved estimation of temporal restoration processes is certainly required and a weakness which affects the entire field focusing on the socio-economic impacts of space weather. For example, Eastwood et al. (2017) make reference to the uncertainty associated with both the spatial and temporal impacts as this can have a dramatic effect on estimated outcomes.
